# Geographic Range of Recreational Water–Associated Primary Amebic Meningoencephalitis, United States, 1978–2018

**DOI:** 10.3201/eid2701.202119

**Published:** 2021-01

**Authors:** Radhika Gharpure, Michelle Gleason, Zainab Salah, Anna J. Blackstock, David Hess-Homeier, Jonathan S. Yoder, Ibne Karim M. Ali, Sarah A. Collier, Jennifer R. Cope

**Affiliations:** Centers for Disease Control and Prevention, Atlanta, Georgia, USA

**Keywords:** Naegleria fowleri, ameba, central nervous system protozoal infections, lakes, rivers, temperature, risk factors, natural resources, United States, protozoa, freshwater, exposures, meningitis/encephalitis, meningoencephalitis, brain infections, primary amebic meningoencephalitis, latitudes, PAM

## Abstract

*Naegleria fowleri* is a free-living ameba that causes primary amebic meningoencephalitis (PAM), a rare but usually fatal disease. We analyzed trends in recreational water exposures associated with PAM cases reported during 1978–2018 in the United States. Although PAM incidence remained stable, the geographic range of exposure locations expanded northward.

Primary amebic meningoencephalitis (PAM) is a rare but usually fatal brain infection caused by *Naegleria fowleri,* a free-living ameba found in soil and warm freshwater (*1*,*2*). The ameba enters the brain via the nasal passages, causing an acute brain infection that usually results in death within 3–7 days of symptom onset. *N. fowleri* is frequently detected in warm freshwater (*3**–**6*); however, <8 of PAM cases are reported each year in the United States (*2*). Generally*,* US PAM cases occur after recreational exposure to warm, untreated freshwater in US southern states during the summer (*2*).

*N. fowleri* is thermophilic, preferring high temperatures of up to 45°C (*7*). However, the ameba tolerates a range of conditions by changing its morphology in response to the environment: it feeds and reproduces in the trophozoite form; assumes a more mobile, flagellated form in low nutrient environments; and forms a resistant cyst in adverse conditions, such as cold temperatures (*1*,*8*). The thermophilic nature of *N. fowleri* suggests it might be sensitive to global changes in surface temperature (*9**–**12*). Analysis of recreational water exposures resulting in PAM could aid risk prediction, prevention, and public communication efforts.

The US Centers for Disease Control and Prevention (CDC) maintains a database of reported PAM cases in the United States since 1962, cataloging information about dates, locations, and suspected exposures (*2*). In 1978, CDC established its Free-living Ameba Laboratory, which increased the national capacity for clinical testing and contributed to an increase in the number of reported PAM cases in the United States. We analyzed to PAM cases reported in the USA during 1978–2018 with known or suspected recreational water exposure in a lake, pond, reservoir, river, stream, or outdoor aquatic venue. 

## The Study

For this study, we included cases with a single known exposure site or multiple sites within an 80 km radius. We conducted negative binomial regression to assess trends in annual PAM incidence. We mapped exposure locations according to regions defined by the United States Census Bureau using ArcMap version 10.5 GIS software (Esri, https://www.arcgis.com). We categorized the exposures into quartiles by case year and evaluated the latitudes of exposure locations using Kruskal-Wallis tests for overall comparisons and Dwass-Steel-Critchlow-Fligner tests for pairwise comparisons. We used linear regression to examine trends in annual maximum (i.e., northernmost) and minimum (i.e., southernmost) latitudes of the exposures. We conducted sensitivity analyses to determine the effect of excluding years with single cases and excluding outliers on the basis of leverage, Cook’s distance, and studentized residual values.

In the temperature analysis, we included patients with a known or imputed date of exposure (Appendix). We obtained temperature records from the National Oceanic and Atmospheric Administration’s National Climatic Data Center (https://www.ncdc.noaa.gov/cdo-web) from the weather station closest to each exposure location (maximum distance of 50 km). We used generalized estimating equation models to compare daily temperatures from the 2 weeks before exposure with average temperatures from the same location and calendar dates from the 20 years before exposure. We selected an autoregressive correlation structure using quasi-likelihood under the independence model criterion. We analyzed data with SAS 9.4 (SAS Institute, https://www.sas.com).

Among 120 PAM cases reported to CDC’s free-living ameba database during 1978–2018, a total of 85 patients had an eligible known or suspected recreational water exposure: 69 patients at a lake, pond, or reservoir; 14 patients at a river or stream; and 2 patients at an outdoor aquatic venue. We excluded 35 patients who were exposed at canals, puddles, or ditches; to geothermally heated water or tap water; at unknown locations; or at multiple locations >80 km apart.

**Figure 1:**
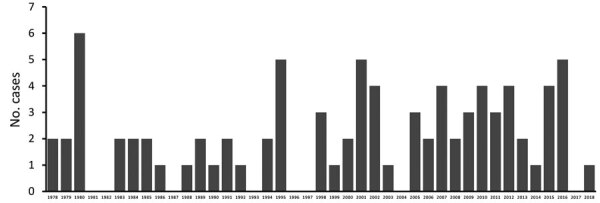
Annual incidence of primary amebic meningoencephalitis cases associated with recreational water exposures, United States, 1978–2018. Negative binomial regression did not detect a trend in the annual incidence of cases (relative risk = 1.015; p = 0.16).

**Figure 2:**
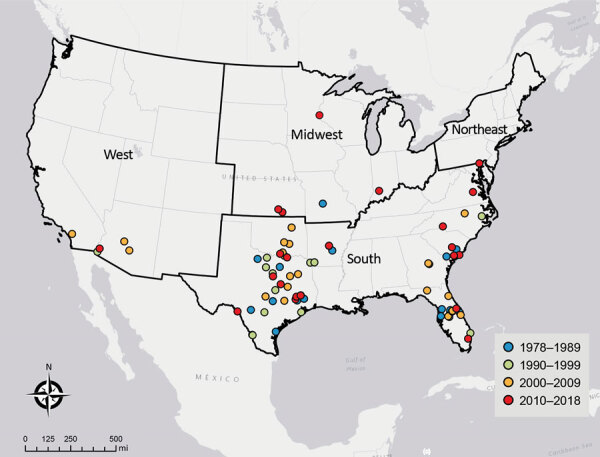
Locations of recreational water exposures associated with cases of primary amebic meningoencephalitis, United States, 1978–2018. Labels indicate US Census Bureau regions.

The incidence of PAM associated with recreational water exposures ranged from 0–6 cases per year during 1978–2018 in the United States (Figure 1). Negative binomial regression did not detect a trend in annual incidence (relative risk [RR] = 1.015; p = 0.16). Among case exposures, 74 occurred in the South and 5 in the West (Figure 2). Six case exposures occurred in the Midwest, 5 of which occurred after 2010 (Minnesota [2010], Kansas [2011], Minnesota [2012], Indiana [2012], and Kansas [2014]).

PAM cases occurred in 33 of the 41 study years. We categorized the case exposures into quartiles by case year: 1978–1989, 1990–1999, 2000–2009, and 2010–2018. The median latitudes of case exposures in the 4 quartiles differed by Kruskal-Wallis analysis (p = 0.02); pairwise comparisons showed an increase in the median latitude of the final quartile, 2010–2018, in comparison to the first quartile, 1978–1989 (p = 0.02) (Table). We modeled the maximum latitude and observed an annual increase of 0.12 decimal degrees (95% CI 0.01–0.20; p = 0.04), equivalent to a shift of ≈13.3 km northward per year. We did not observe a change in the minimum latitude (0.04 decimal degrees, 95% CI −0.04 to 0.10; p = 0.30). We used sensitivity analyses to confirm a northward trend in maximum latitude (Appendix).

**Table:** Recreational water–associated primary amebic meningoencephalitis, United States, 1978–2018

Years	No. cases	Median latitude, decimal degrees (range)	p value*
1978–1989	20	30.3 (27.8–38.0)	Referent
1990–1999	15	32.5 (26.5–35.5)	0.90
2000–2009	26	32.3 (28.1–36.1)	0.47
2010–2018	24	33.6 (26.1–45.0)	0.02

*Pairwise comparison to reference group using Dwass-Steel-Critchlow-Fligner test.

Among 85 cases, 81 (95%) had a known or imputed exposure date. Air temperatures varied widely in the 2 weeks before exposure (Appendix Figure). On average, daily temperatures were higher in the 2 weeks before exposure than the 20-year average for that date and location: high temperatures were 0.77°C greater (p<0.01), and low temperatures were 0.76°C greater (p<0.01) than the 20-year average.

## Conclusions

The rise in cases in the Midwest region after 2010 and increases in maximum and median latitudes of PAM case exposures suggest a northward expansion of *N. fowleri* exposures associated with lakes, ponds, reservoirs, rivers, streams, and outdoor aquatic venues in the United States. We observed an increase in air temperatures in the 2 weeks before exposures compared with 20-year historic averages. It is possible that rising temperatures and consequent increases in recreational water use, such as swimming and water sports, could contribute to the changing epidemiology of PAM. Although reported incidence of PAM has increased worldwide (*12*,*13*), the incidence of reported cases of PAM in the United States remained stable during 1978–2018. The worldwide trends might reflect changes in international diagnostic capacity (*13*).

This study is subject to limitations. First, PAM is probably underrecognized and underreported in the United States (*14*), so these data might not fully capture trends in incidence and exposure characteristics. Second, temperature data were not collected simultaneously with exposure, and thus might differ from actual exposure conditions. Our analysis used air temperatures because water temperature records were unavailable for most exposure sites. However, prior studies have indicated that air temperature is the main driver for lake surface temperatures (*15*) and thus is an appropriate proxy. Third, our analysis included years with single cases, which could bias the results of the regression analyses of latitude. However, our sensitivity analysis indicated that these years did not change our findings. 

In summary, our results show a suggested northward expansion of PAM and its potential association with higher temperatures warrants further investigation. Characterizing recreational water exposures could improve risk prediction and prevention strategies, helping to prevent cases, aid natural resource custodians, and reduce burden on state and local health departments.

Appendix Additional information on imputation methods, sensitivity analyses, and air temperature trends related to primary amebic meningoencephalitis cases associated with recreational water exposures, United States, 1978–2018.
