# Supplementary material for: Geographic Range of Recreational Water–Associated Primary Amebic Meningoencephalitis, United States, 1978–2018
Source: Emerg Infect Dis. 2021 Jan;27(1):271–4. doi: 10.3201/eid2701.202119 (PMC7774533; doi:10.3201/eid2701.202119)
Supplement: Appendix — Additional information on imputation methods, sensitivity analyses, and air temperature trends related to primary amebic meningoencephalitis cases associated with recreational water exposures, United States, 1978–2018. [file 20-2119-Techapp-s1.pdf]

Article DOI: https://doi.org/10.3201/eid2701.202119

Geographic Range of Recreational Water-Associated Primary Amebic Meningoencephalitis, United States, 1978–2018

Appendix

Case Exposure Date Imputation Methods for Temperature Analyses

If the exposure date was known, a case was included without further changes (n = 32). If a case did not have a recorded exposure date or had multiple possible exposure dates, the following imputation method was used: if date of death was known (n = 36), the case was assigned a date of exposure using the average number of days between exposure and death (10.0 days) of cases with known dates in the database. Otherwise, if date of onset was known (n = 6), the case was assigned a date of exposure using the average number of days between exposure and onset (5.3 days) of cases with known dates in the database. If the dates of onset, exposure, and death were all unknown but month of exposure was known (n = 7), a day of the month was assigned using a random number generator; for cases with known exposure dates, exposure day during the month was roughly evenly distributed during each month.

Results of Sensitivity Analyses for Trends in Latitude

When years with single cases were removed from analysis, the maximum latitude increased by 0.13 decimal degrees per year (95% CI –0.0004 to 0.3, p = 0.06), equivalent to 14.4 km northward, and when years with outlier data were removed, the maximum latitude increased by 0.07 decimal degrees per year (95% CI –0.02 to 0.2, p = 0.11), equivalent to 7.7 km northward.

When years with single cases were removed from analysis, the minimum latitude increased by 0.03 decimal degrees per year (95% CI –0.04 to 0.1, p = 0.42) and when years with outlier data were removed, the minimum latitude increased by 0.03 decimal degrees per year (95% CI –0.05 to 0.1, p = 0.49).

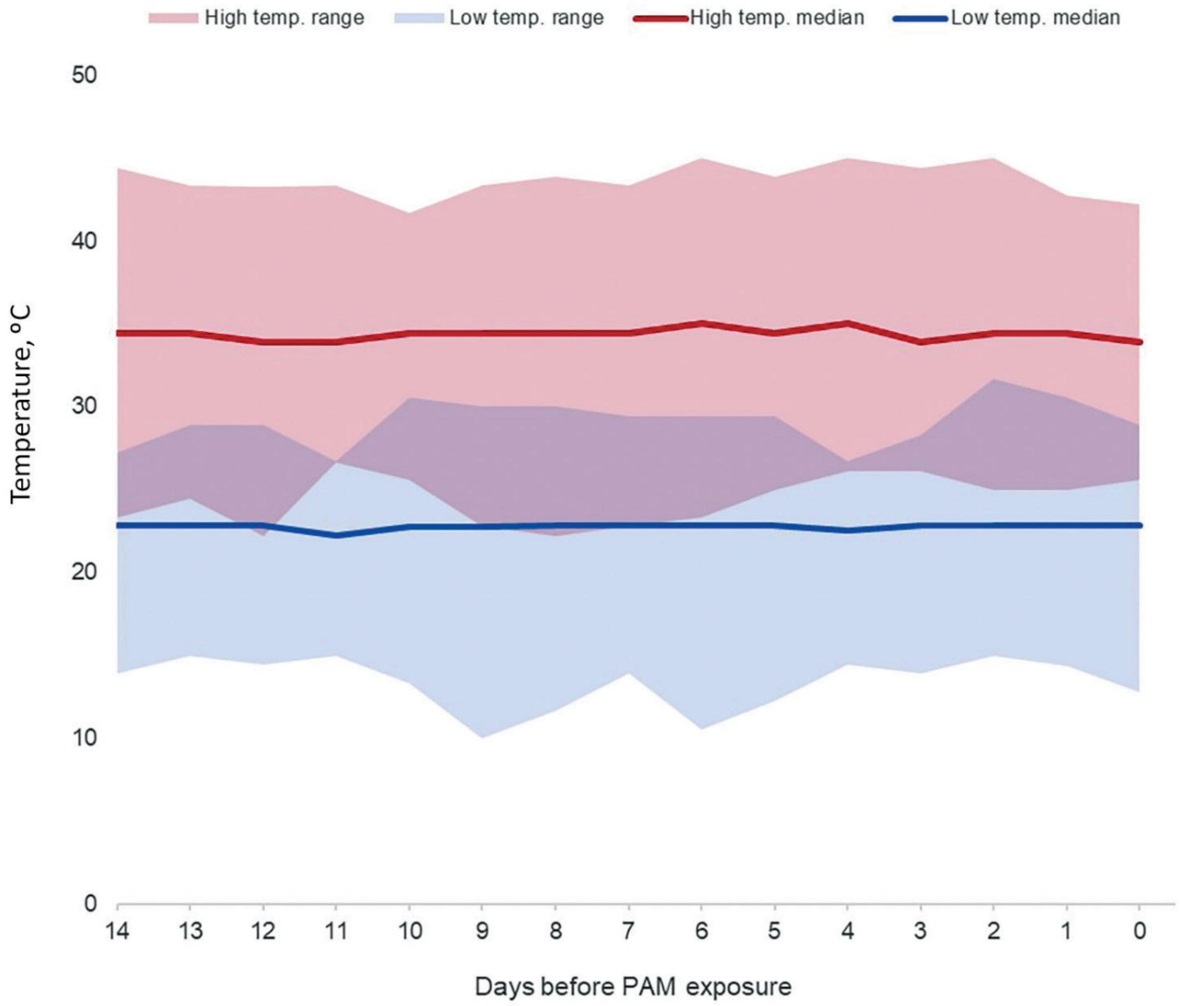

Appendix Figure. Air temperatures in the 2 weeks before recreational water exposures associated with cases of primary amebic meningoencephalitis, United States, 1978–2018. Figure shows cases with known or imputed date of exposure.
